# The Neuronal Ischemic Tolerance Is Conditioned by the *Tp53 Arg72Pro* Polymorphism

**DOI:** 10.1007/s12975-018-0631-1

**Published:** 2018-04-23

**Authors:** Maria E. Ramos-Araque, Cristina Rodriguez, Rebeca Vecino, Elisa Cortijo Garcia, Mercedes de Lera Alfonso, Mercedes Sanchez Barba, Laura Colàs-Campàs, Francisco Purroy, Juan F. Arenillas, Angeles Almeida, Maria Delgado-Esteban

**Affiliations:** 1 Institute of Biomedical Research of Salamanca, University Hospital of Salamanca, University of Salamanca, CSIC, Calle Zacarías González 2, 37007 Salamanca, Spain; 2 Institute of Functional Biology and Genomics, University of Salamanca, CSIC, Salamanca, Spain; 3 Stroke Unit, Department of Neurology, University Hospital of Valladolid, University of Valladolid, Valladolid, Spain; 4 Department of Statistics, University Hospital of Salamanca, University of Salamanca, Salamanca, Spain; 5 Clinical Neurosciences Group, IRBLleida. UdL, Lleida, Spain; 6 Stroke Unit, University Hospital Arnau de Vilanova, Lleida, Spain; 7 Neurovascular Research Laboratory (i3), Instituto de Biología y Genética Molecular, Universidad de Valladolid, CSIC, Valladolid, Spain

**Keywords:** Ischemic tolerance, Preconditioning, *Tp53 Arg72Pro* polymorphism, Neuroprotection, Transient ischemic attack

## Abstract

Cerebral preconditioning (PC) confers endogenous brain protection after stroke. Ischemic stroke patients with a prior transient ischemic attack (TIA) may potentially be in a preconditioned state. Although PC has been associated with the activation of pro-survival signals, the mechanism by which preconditioning confers neuroprotection is not yet fully clarified. Recently, we have described that PC-mediated neuroprotection against ischemic insult is promoted by p53 destabilization, which is mediated by its main regulator MDM2. Moreover, we have previously described that the human *Tp53 Arg72Pro* single nucleotide polymorphism (SNP) controls susceptibility to ischemia-induced neuronal apoptosis and governs the functional outcome of patients after stroke. Here, we studied the contribution of the human *Tp53 Arg72Pro* SNP on PC-induced neuroprotection after ischemia. Our results showed that cortical neurons expressing the Pro72-p53 variant exhibited higher PC-mediated neuroprotection as compared with Arg72-p53 neurons. PC prevented ischemia-induced nuclear and cytosolic p53 stabilization in Pro72-p53 neurons. However, PC failed to prevent mitochondrial p53 stabilization, which occurs in Arg72-p53 neurons after ischemia. Furthermore, PC promoted neuroprotection against ischemia by controlling the p53/active caspase-3 pathway in Pro72-p53, but not in Arg72-p53 neurons. Finally, we found that good prognosis associated to TIA within 1 month prior to ischemic stroke was restricted to patients harboring the Pro72 allele. Our findings demonstrate that the *Tp53 Arg72Pro* SNP controls PC-promoted neuroprotection against a subsequent ischemic insult by modulating mitochondrial p53 stabilization and then modulates TIA-induced ischemic tolerance.

## Introduction

The restriction in blood supply to the brain causes a shortage of oxygen and glucose to maintain cellular metabolism, which elicits a pathological response. Paradoxically, transient brief episodes of controlled ischemia confer protection against a subsequent prolonged ischemia. This phenomenon described as cerebral preconditioning (PC) has been evidenced both in vitro and in vivo models [1–3]. The efficacy of PC in reducing brain injury after an ischemic damage has also been demonstrated in clinical studies. Transient ischemic attack (TIA) is considered to be the clinical correlate of PC. Thus, patients with previous ipsilateral TIA before an ischemic stroke had better outcome than patients without previous TIA [4–6]. Therefore, it provides clear evidence that PC may be effective to reduce brain injury in human against ischemic insult [7–10].

In recent years, there has been increasing interest in the study of molecular mechanisms responsible for PC-mediated neuroprotection, as well as its possible application in therapies for cerebral ischemic damage [1, 2]. It was reported that cerebral PC is implicated in different mechanisms such as the inhibition of glutamate release [11, 12], or the increase in neuronal resistance to the excitotoxic insult [13–15]. This neuroprotective response initiated through the post-translational modification of proteins is rapid and occurs within minutes to hours [16–18]. However, the molecular mechanisms responsible for PC-mediated neuroprotection have not been elucidated so far.

The deletion of the *Tp53* gene is associated with neuroprotection following ischemia [19]. Moreover, since nuclear p53 transcriptional activity has been associated specifically with apoptosis of cortical neurons [20], the interference with p53 activity has inspired potential applications in therapy. The protein p53 is translocated from the cytosol to the mitochondria and mediates transcriptional dependent or independent apoptosis cell death [21, 22], in response to stimulus such as ischemia [20]. In this context, several studies have shown that p53 is translocated from the cytosol to the mitochondria [23, 24] in response to ischemia. Indeed, p53 mediates apoptosis through its direct action in the mitochondria, where p53 binds to pro-apoptotic PUMA and Bax, promoting cytochrome c release [25], in different cell types and tissues, including brain [22, 26]. In contrast, the inhibition of caspase-3-mediated apoptotic pathway activation by PC has also been associated with ischemic tolerance in transient cerebral ischemia in vivo models [27]. Accordingly, several studies have shown that PC decreases neuronal apoptosis and reduces the subsequent neurological deficits and infarct volume within 24 to 72 h after ischemia [28, 29], by promoting ischemic tolerance [27]. In a recent work, we have demonstrated that PC confers neuronal ischemic tolerance by increasing MDM2 protein level, which promotes its interaction with p53 and triggers p53 nuclear and cytosolic destabilization [30]. Indeed, our previous results have demonstrated that PC attenuates ischemia-induced activation of the p53/PUMA/caspase-3 signaling pathway and subsequent neuronal death. Hence, the aim of our study was to identify the function of p53 and related proteins on neuroprotection associated to PC prior to a later ischemic insult and its role in neuronal ischemic tolerance.

Previously, we have identified that the human *Tp53 Arg72Pro* polymorphism explains different functional prognosis in stroke [22, 31]. We found that Arg72-p53, but not Pro72-p53 polymorphic variant, interacts directly with Bcl-xL in the mitochondria, which increases neuronal vulnerability to ischemia-induced apoptotic cell death [22]. In addition, our group have recently published that the *Tp53 Arg72Pro* SNP determines neovascularization, brain repair, and neurological recovery after intracerebral hemorrhage [31]. Here, we study the function of human *Tp53 Arg72Pro* SNP on PC-induced neuroprotection and ischemic tolerance. Our results show that PC prevented ischemia-induced nuclear and cytosolic p53 stabilization in Pro72-p53; however, this effect was not observed in Arg72-p53, cortical neurons. Moreover, PC prevented ischemia-induced neuronal apoptosis and caspase-3 activation, leading to ischemic tolerance in Pro72-p53 cortical neurons. Hence, here, we aim to report for the first time that the *Tp53 Arg72Pro* SNP modulates PC-induced neuroprotection against ischemia by controlling the p53/caspase-3 signaling pathway. Moreover, we aim to confirm the relevance of the *Tp53 Arg72Pro* SNP in functional outcome of ischemic stroke patients within the first month after a transitory ischemic attack (TIA).

## Methods

### Primary Cultures of Cortical Neurons

Neuronal cultures were prepared from E14.5 mouse embryo cortices. The humanized knock-in mice for the *Tp53 Arg72Pro* SNP were kindly donated by Prof D.G. Johnson (The University of Texas MD Anderson Cancer Center, Smithville, Texas, USA) [32]. Animals were maintained in specific-pathogen-free facilities at the University of Salamanca, in accordance with Spanish legislation (RD53/2013) under license from the Spanish government and the EU (2010/63/EU). Protocols were approved by the Bioethics Committee of the University of Salamanca. Neurons expressing two different polymorphic variants (Pro72-p53 neurons or Arg72-p53 neurons) were seeded at 1.8 × 10^5^ cells/cm^2^ in Neurobasal medium (Invitrogen, Madrid, Spain) supplemented with 2% B27 (Invitrogen) and glutamine 2 mM (Invitrogen), and incubated at 37 °C in a humidified 5% CO_2_-containing atmosphere. Half of the culture medium was replaced with fresh medium every 3–4 days.

### Oxygen Glucose Deprivation and Preconditioning Models

After 9–10 DIV, neurons were exposed to oxygen and glucose deprivation (OGD) induced by incubating cells at 37 °C for 90 min in an incubator equipped with an air lock and continuously gassed with 95% N_2_/5% CO_2_ (Thermo Scientific Forma Water Jacket CO_2_ incubator); with a CO_2_ infrared sensor and variable oxygen control, connecting with injection of N_2_ gas supply to control oxygen at suppressed levels and a fuel cell as oxygen control sensor that puts out a linear millivolt signal based on O_2_ content of the chamber and provides fast recovery to desired hypoxic conditions for reliable and accurate control. The incubation medium (buffered Hanks’ solution without glucose: 5.26 mM KCl, 0.43 mM KH_2_PO_4_, 132.4 mM NaCl, 4.09 mM NaHCO_3_, 0.33 mM Na_2_HPO_4_, 2 mM CaCl_2_, and 20 mM HEPES, pH 7.4) was previously gassed with 95% N_2_/5% CO2 for 30 min. Under these conditions, oxygen concentrations in the incubation medium were 6.7 ± 0.5 μM as measured with a Clark-type oxygen electrode [33, 34]. In case of preconditioning (PC), the neurons were treated with a moderated subtoxic concentration of N-Methyl-d-Aspartate (20 μM, NMDA-PC) for 2 h prior to a subsequent lethal ischemia induced by oxygen and glucose deprivation (OGD; 90 min); (NMDA-PC + OGD) previously validated PC method [30, 35, 36]. In parallel, neurons were incubated under Normoxia (Nx) or NMDA-PC conditions at 37 °C in a humidified atmosphere of 95% air/5% CO_2_ in buffered Hanks’ solution buffer. Under these conditions, oxygen concentrations in the incubation medium were 190 ± 15 μM as measured with a Clark-type oxygen electrode. Unless otherwise stated, neuronal samples were collected at 4 h of reoxygenation after OGD and processed accordingly.

### Flow Cytometric Detection of Apoptotic Cell Death

Neurons were carefully detached from the plates using 1 mM EDTA (tetrasodium salt) in PBS (pH 7.4) at room temperature. Cells were stained with annexin V-APC and 7-AAD in binding buffer (100 mM HEPES, 140 mM NaCl, 2.5 mM CaCl_2_), according to the manufacturer’s instructions and performed exactly as we previously described [22], to determine quantitatively the percentage of apoptotic neurons by flow cytometry. Four replicates per condition (3 × 10^5^ cells each) were analyzed on a FACScalibur flow cytometer (15 mW argon ion laser tuned at 488 nm; CellQuest software, BD Biosciences). The annexin V-APC stained cells that were 7AAD negative were considered to be apoptotic (AnnexinV^+^/7AAD^−^).

### Flow Cytometric Analysis of Membrane Potential (Ψm)

Ψm was assessed using the MitoProbe DilC1(5) Assay Kit for Flow Cytometry (Invitrogen), and stained cells were analyzed on the FL1 and FL4 channels of a FACScalibur flow cytometer (15 mW argon ion laser tuned at 488 nm; CellQuest software; BD Biosciences) as we previously described [22]. Ψm values were expressed as percentages, and the 10 μM of mitochondrial uncoupler carbonyl cyanide 4-(trifluoromethoxy) phenylhydrazone (10 μM; FCCP) was used for 15 min to define the 0% Ψm values.

### Quantitative Reverse Transcription-Polymerase Chain Reaction (RT-qPCR) Analysis

Total RNA samples were purified from neurons using a commercially available kit (Sigma) and RT-qPCR was performed with Power SYBR Green RNA-to-CT TM 1-Step kit (Applied Biosystems, Township, USA). Reverse transcription was performed for 30 min at 48 °C, and PCR conditions were 10 min at 95 °C followed by 40 cycles of 15 s at 95 °C plus 1 min at 55 °C using the following forward and reserve primers, respectively (Thermo Scientific, Offenbach, Germany), 5′-ATTCTGCCCACCACACAGCGACA-3′ and 5′ AGGGCTTCCTCTGGGCCTTCTA-3′ (p53), 5′-GGGTGTGAACCACGAGAAAT-3′ and 5′-GACTGTGGTCATGAGCCCTT-3′ (Gapdh). The mRNA abundance of each transcript was normalized to the Gapdh mRNA abundance obtained in the same sample. The relative mRNA levels were calculated using the ΔΔCt method and were expressed as the fold change between sample and calibrator.

### Caspase-3 Activity Assay

Caspase-3 activity was assessed in cell lysates and according to manufacturers’ instructions via Fluorimetric Assay kit CASP3F from SIGMA and read at emission wavelength 405 nm. The method is based on the release of the fluorescent 7-amino-4-methylcoumarin (AMC) moiety. The AMC concentration is calculated using a AMC standard.

### Western Blotting

Neurons were lysed in buffer containing 1% SDS, 2 mM EDTA, 150 mM NaCl, 12.5 mM Na_2_HPO_4_, and 1% Triton X-100 (NP40: 1% NP40, EDTA diK^+^ 5 mM, Tris pH 8 20 mM, NaCl 135 mM, and 10% glycerol), supplemented with phosphatase inhibitors (1 mM Na_3_VO_4_ and 50 mM NaF) and protease inhibitors (100 μM phenylmethylsulfonyl fluoride, 50 μg/ml anti-papain, 50 μg/ml pepstatin, 50 μg/ml amastatin, 50 μg/ml leupeptin, 50 μg/ml bestatin, and 50 μg/ml soybean trypsin inhibitor), stored on ice for 30 min, and boiled for 5 min. Protein concentrations were determined with the BCA (bicinchoninic acid) method, using bovine serum albumin as a standard (BCA Protein Assay kit, Thermo Fisher Scientific). Extracts were centrifuged at 13,000g for 5 min at 4 °C. Aliquots of lysed, nuclear, cytosolic, or mitochondrial extracts (20–70 μg protein) were subjected to SDS-polyacrylamide gel (Mini PROTEAN; Bio-Rad Laboratories) and blotted with anti-p53 (1:100) (554157, BDBiosciences), anti-cleaved caspase-3 (Asp175, 9661, Cell Signaling), anti-MDM2 (2A10, ab16895), anti-PUMA (ab54288) (Abcam, Cambridge, UK), and anti-lamin B (sc-374015, Santa Cruz Biotechnology, Heidelberg, Germany) anti-GAPDH (Ambion, Cambridge, UK) overnight at 4 °C. Signal detection was performed with an enhanced chemiluminescence detection kit (Thermo Fisher Scientific), and the autoradiograms were scanned. Band intensities were quantified using ImageJ software [37].

### Subcellular Fractionation

Cells were washed with cold PBS containing 1 mM MgCl_2_, harvested with cytosolic buffer (10 mM HEPES, 1.5 mM MgCl_2_, 10 mM KCl, 1 mM EDTA, 0.1% NP-40, *v*/*v*, 1.5 M sucrose, and protease and phosphatase inhibitors mixture, pH 7.9), triturated with a micropipette to promote cell lysis, left on ice for 30 min, and vortexed for 10 s. After checking cell lysis under a light microscope, extracts were centrifuged at 830×*g* for 10 min. Lysis of the nuclei was performed by resuspending the nuclear pellet in nuclear buffer (50 mM HEPES, 1.5 mM MgCl_2_, 10 mM KCl mM, 0.5 mM NaCl, 1 mM EDTA, 1% NP-40, *v*/*v*, and protease and phosphatase inhibitor mixture, pH 7.9), triturated with a micropipette, left on ice for 2 h, vortexed (10 s), boiled (5 min), and sonicated (5 min). The supernatant (mitochondrial and cytosolic fractions) was then centrifuged at 17,000×*g* for 12 min (4 °C), and the cytosolic fraction (supernatant) was lysed in 2× RIPA buffer (2% sodium dodecyl sulfate, 2 mM EDTA, 2 mM EGTA, and 50 mM Tris, pH 7.5), supplemented with phosphatase inhibitors (1 mM Na_3_VO_4_ and 50 mM NaF) and protease inhibitors (100 μM phenylmethylsulfonyl fluoride (PMSF), 50 μg/ml anti-papain, 50 μg/ml pepstatin, 50 μg/ml amastatin, 50 μg/ml leupeptin, 50 μg/ml bestatin, and 50 μg/ml soybean trypsin inhibitor), and boiled for 5 min [37]. The mitochondrial fraction (pellet) was resuspended in isolation medium (320 mM sucrose, 1 mM potassium EDTA, and 10 mM Tris-HCl, pH 7.4) and was homogenized in a tight-fitting glass—Teflon homogenizer (20 strokes) [22, 38]. Mitochondrial fraction was lysed with 2× RIPA buffer for protein analysis by immunoblotting.

### Immunocytochemistry

Neurons grown on glass coverslips were fixed with 4% (*v*/*v*, in PBS) paraformaldehyde for 30 min and immunostained with rabbit anti-cleaved caspase3 (Asp175) (1:300; Cell Signaling Techn, Inc.), mouse anti-MAP2 (1:500; SIGMA) [39], mouse anti-p53 (1:200; 554157, BD Biosciences) [30], and rabbit-COX IV (1:1000; ab16056, Abcam) antibodies. Immunolabeling was detected using anti-rabbit IgG–Cy3 (1:500) or anti-mouse IgG–Cy2 (1:500; Jackson ImmunoResearch). Coverslips were washed, mounted in SlowFade light anti-fade reagent (Invitrogen) on glass slides, and examined using a microscope (Nikon Inverted microscope Eclipse Ti-E) equipped with × 20 objective and a pre-centered fiber illuminator Nikon Intensilight C-HGFI and black and white charge-coupled device digital camera Hamamatsu ORCAER, or on a scanning laser confocal microscope (“Spinning disk” Roper Scientific Olympus IX81) with three lasers (405, 491, and 561 nm) and equipped with × 40 and × 63 PL Apo oil-immersion objective for high resolution imaging and device digital camera Evolve Photometrics.

### Patient Cohorts

Nested case control study was designed on previously collected cohorts of patients with ischemic stroke defined as infarction of the central nervous system tissue [40], between 2012 and 2015 in three University Hospitals (Salamanca, Valladolid, and Lleida, Spain). Among this cohort, we selected patients with prior TIA defined as a brief episode of neurological dysfunction caused by focal brain or retinal ischemia, with clinical symptoms typically lasting less than 1 h, and without evidence of acute infarction [40] and a randomized subset of patients without it. Inclusion criteria were acute ischemic stroke patients, previously independent for daily life activities, with a TIA within 1 month before the stroke, and patients without it. Patients were admitted within the first 24 h after the onset of symptoms, or from the start of sleep in those with symptoms upon awakening. Exclusion criteria were intracerebral hemorrhage, TIA history more than 1 month before the stroke, insufficient clinical data, and lack of informed consent. Eighty-five patients fulfilled all inclusion criteria for the study. Clinical and epidemiological data were taken from patients’ charts. The study was approved by the local ethics committees. All the patients or their relatives provided their informed consent before taking part of the study.

### Clinical Variables

Patients were admitted to the Neurology departments and treated according to the Guidelines of the cerebrovascular disease study Group of the Spanish Society of Neurology [41]. Baseline demographic and clinical data included age, sex, pre-onset modified Rankin Scale (mRS), past medical history, vascular risk factors, and previous treatment. Standard 12-lead ECG and cranial Computed Tomography (CT) or brain Magnetic Resonance (MR) were performed at admission. Details of previous TIA were obtained from the patient and medical records. Standard definitions of TIA and stroke were used. Stroke subtypes were classified according to the TOAST criteria [42]. Stroke severity was assessed using the National Institute of Health Stroke Scale (NIHSS) at admission. Functional outcome was evaluated at 3 months after stroke using the mRS. NIHSS and mRS were evaluated by internationally certified neurologists. The main outcome measure for all patients was functional status at 3 months; mRS score ≤ 2 was considered good outcome [43].

### Human *Tp53* Polymorphism Analysis

Genotyping of human *Tp53* polymorphism was performed at the University of Salamanca by authors blinded to the clinical status of patients, by using the PCR-RFLP technique [22]. The *Tp53* SNP of exon 4 at codon 72 (*Arg72Pro*; rs1042522) was detected by amplifying genomic DNA with the forward primer 5′-TCTACAGTCCCCCTTGCCGT-3′ and the reverse primer 5′-CTGACCGTGCAAGTCACAGA-3′. *Tp53* exon 4, where BstU1 (Bsh1236I) RFLP is located, was amplified within a 298-bp DNA fragment. Digests were separated on agarose gel (3%) and the Midori Green Advance-stained DNA fragments (Nippon Genetics Europe GmbH, Düren, Germany) were analyzed under a UV source using the Bio-Rad Universal Hood II Gel Imager system (Bio-Rad, CA, USA). The distribution of genotype frequencies in *Arg72Pro* between the stroke patients was within the Hardy–Weinberg equilibrium (*p* > 0.1 in all cases). As the *Pro* allele is likely to exert a dominant effect on the *Arg* one [22], two groups were considered for this study: Pro (*Pro*/*Pro* and *Pro*/*Arg* genotypes) and Arg (*Arg*/*Arg* genotype) patients.

### Statistical Analysis

The results obtained in cell cultures are expressed as means ± SEM values from four different culture preparations. Statistical analyses were performed by one-way analysis of variance, followed by the least significant difference multiple range test. In these cases, *p* < 0.05 value was considered significant. Clinical results are expressed as percentages for categorical variables. For the continuous variables, the results are expressed as either the mean ± S.D. or median (25th and 75th percentiles) depending on whether or not the data followed a normal distribution, respectively. The Kolmogorov–Smirnov test was used for testing the normality of the distribution. The Student *t* test (normal data) or the Mann–Whitney test (non-normal data) was used to compare continuous variables between two groups. Proportions were compared using the *x*^2^ and fisher test. In all instance, *p* < 0.05 values were considered significant. Statistical analyses were performed using SPSS Statistics 22.0 for Macintosh (IBM, Madrid, Spain).

## Results

### NMDA-PC Prevents Ischemia-Induced p53 Stabilization in Pro72-p53 Neurons, but Not in Arg72-p53 Neurons

**Fig. 1:**
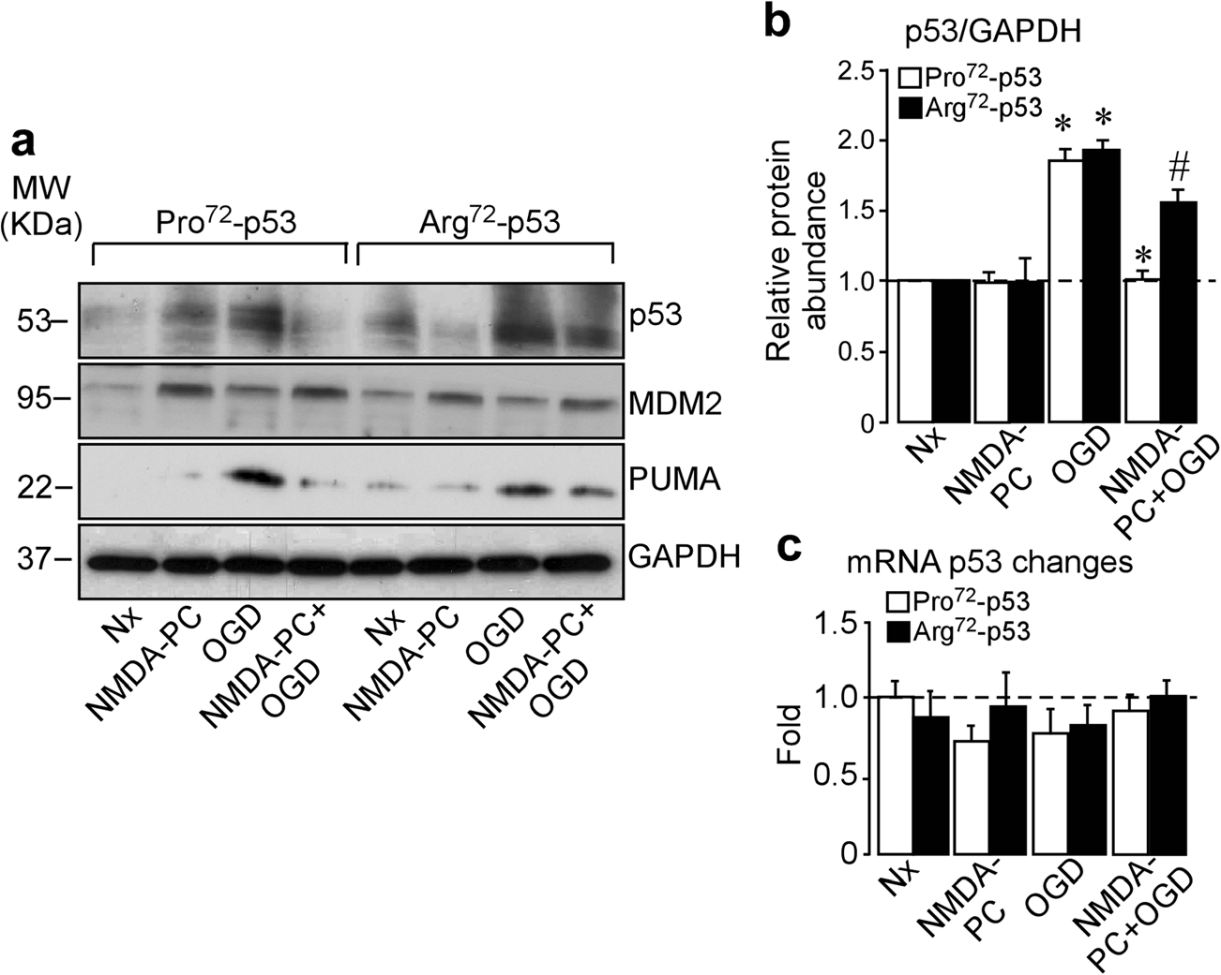
NMDA-PC promotes p53 destabilization in Pro72-p53 neurons. Cortical neurons (9–10 DIV) obtained from humanized knock-in mice for the both codon Arg72Pro variants of p53 (Pro72-p53 and Arg72-p53) were treated with a moderate subtoxic N-Methyl-d-Aspartate (20 μM) for 2 h prior to OGD (NMDA-PC+OGD), a validated in vitro model of PC and p53 and its related protein expression, (**a**) neuronal extracts were analyzed by Western blotting and (**b**) relative protein abundance of p53 was quantified. (**a**, **b**) OGD induced p53 stabilization and its target PUMA at 4 h after OGD, which was prevented by NMDA-PC only in Pro72-p53 neurons, as revealed by Western blotting. Furthermore, as shown in (**a**) similar expression levels of its E3 ubiquitin ligase, MDM2 were found in neurons expressing both Arg72Pro variants of p53, after OGD and NMDA-PC conditions. GADPH protein levels were used as loading control. A representative Western blot is shown out of four. (**c**) RT-qPCR analysis of p53 gene reveals that p53 mRNA remained unaltered after OGD in neurons expressing for both *Arg72Pro* variants of p53. Data are means ± S.E.M. (*n* = 4 independent neuronal cultures). Statistical analysis of the results was evaluated by one-way analysis of variance, followed by the least significant difference multiple range test. Student’s *t* test was used for comparisons between two groups of values. In all cases, *p* < 0.05 value was considered significant.*p < 0.05 versus Nx. #*p* < 0.05 versus OGD

We have previously identified that the Arg72-p53, but not Pro72-p53, interacts directly with anti-apoptotic mitochondrial Bcl-xL, then increasing vulnerability to ischemia-induced apoptotic death in cortical neurons [22]. To study the role of human *Tp53 Arg72Pro* SNP on the PC-mediated neuroprotection, we obtained cortical neurons from humanized *Tp53* knock-in mice for the *Arg72Pro* SNP. First, neurons expressing human Pro72-p53 or Arg72-p53 variants were exposed to a validated in vitro model of PC induced by NMDA (NMDA-PC) [30, 36] and/or oxygen glucose deprivation (OGD) protocol [33]. As shown in Fig. 1a, b, 4 h of reoxygenation following OGD induced p53 stabilization and the expression of its transcriptional target PUMA in both Pro72-p53 and Arg72-p53 neurons, when compared with normoxic (Nx) conditions. These results demonstrate that neurons expressing either Pro72-p53 or Arg72-p53 are equally sensitive to ischemia-mediated p53 stabilization. However, NMDA-PC prior to the insult (OGD) prevented p53 stabilization in Pro72-p53 neurons, but not in neurons expressing the Arg72-p53 SNP variant (Fig. 1a, b). Since NMDA-PC increased MDM2 levels in neurons (Fig. 1a) and this effect was maintained after OGD in both genotypes, we therefore confirmed that only preconditioned Pro^72^-p53 neurons might display p53 destabilization after ischemia through a MDM2-dependent mechanism. According to our previous results [30], we also verified that p53 mRNA remained unaltered during preconditioning (Fig. 1c), which highlights the importance of post-translational regulation of p53 after ischemic events. These results demonstrate that human *Tp53 Arg72Pro* polymorphism conditions NMDA-PC-mediated p53 destabilization after OGD, which might control neuronal susceptibility to the ischemic insult.

**Fig. 2:**
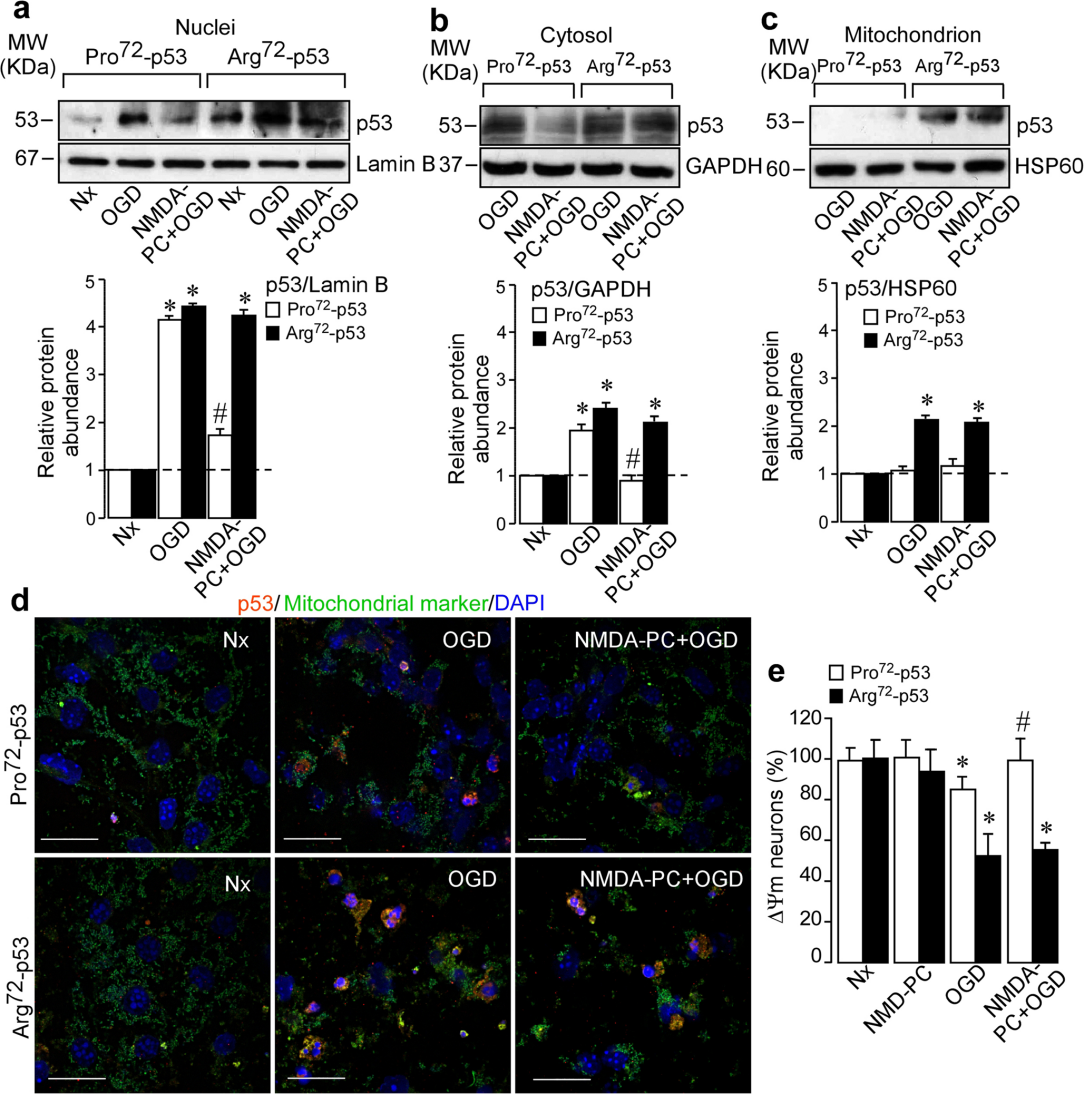
NMDA-PC failed to prevent mitochondrial p53 destabilization in Arg72-p53 neurons. Cortical neurons from mice expressing human Pro72-p53 or Arg72-p53 variant (9–10 DIV) were exposed to a validated in vitro model of PC. NMDA-PC promoted p53 destabilization levels at 4 h after OGD, only in Pro72-p53 neurons. This effect occurred in both nuclei (**a**) and cytosol (**b**), which confirmed that NMDA-PC prevented the accumulation of p53 induced by OGD only in Pro72-p53 neurons. Lamin B and GAPDH protein levels were used as nuclear and cytosolic loading control, respectively. (**c**) OGD induced mitochondria p53 stabilization in Arg72-p53 neurons but not in Pro72-p53 neurons, as assayed by Western blotting analysis. HSP60 protein levels were used as loading control. A representative Western blot is shown out of four. (**d**) This effect was corroborated after co-immunostaining for anti-p53 (red) and mitochondrial marker anti-COX IV (green). Scale bar 20 μm. (**e**) OGD decreased mitochondria membrane potential (Ψm) by 18% in Pro72-p53 neurons and 50% in Arg72-p53 neurons. Nevertheless, NMDA-PC prevented OGD-induced mitochondria membrane potential decreased in Pro72-p53 neurons, but not in Arg72-p53 neurons, at 4 h after OGD. Data are means ± S.E.M. Statistical analysis of the results was evaluated by one-way analysis of variance, followed by the least significant difference multiple range test. Student’s *t* test was used for comparisons between two groups of values. In all cases, *p* < 0.05 value was considered significant. In all cases, *p < 0.05 versus Nx and #*p* < 0.05 versus OGD

To transactivate p53 target genes such as p21, PUMA, and p53 itself, the stabilization and subsequent nuclear translocation of p53 is required [44, 45]. Thus, we investigated whether NMDA-PC prevents ischemia-induced p53 nuclear translocation. Nuclear, cytosolic and mitochondrial neuronal fractions were obtained separately, and protein levels were analyzed by Western blotting. As shown in Fig. 2a, p53 is translocated to the nucleus in both Pro72-p53 and Arg72-p53 neurons after reoxygenation following ischemia. However, the nuclear (Fig. 2a) and cytosolic (Fig. 2b) destabilization of p53 induced by NMDA-PC was evidenced only in Pro72-p53 neurons at 4 h after OGD. In the case of Arg72-p53 neurons, NMDA-PC failed to prevent ischemia-induced mitochondrial p53 accumulation, as shown after Western blot analysis (Fig. 2c) and co-immunostaining with anti-p53 and mitochondrial marker (Fig. 2d), respectively. Moreover, preconditioned neurons expressing Pro72-p53 showed intact mitochondrial membrane potential (Δψ_m_) in contrast to those expressing Arg72-p53, which collapsed their Δψ_m_ (Fig. 2e). Hence, Arg72-p53 cortical neurons are more susceptible to p53-associated mitochondria dysfunction than Pro72-p53 neurons, as we previously described [22]. Moreover, NMDA-PC failed to prevent p53 stabilization after ischemia in neurons expressing the Arg72-p53 variant.

### NMDA-PC Prevents Ischemia-Induced Apoptosis and Caspase-3 Activation in Pro72-p53 Neurons, but Not in Arg72-p53 Neurons

**Fig. 3:**
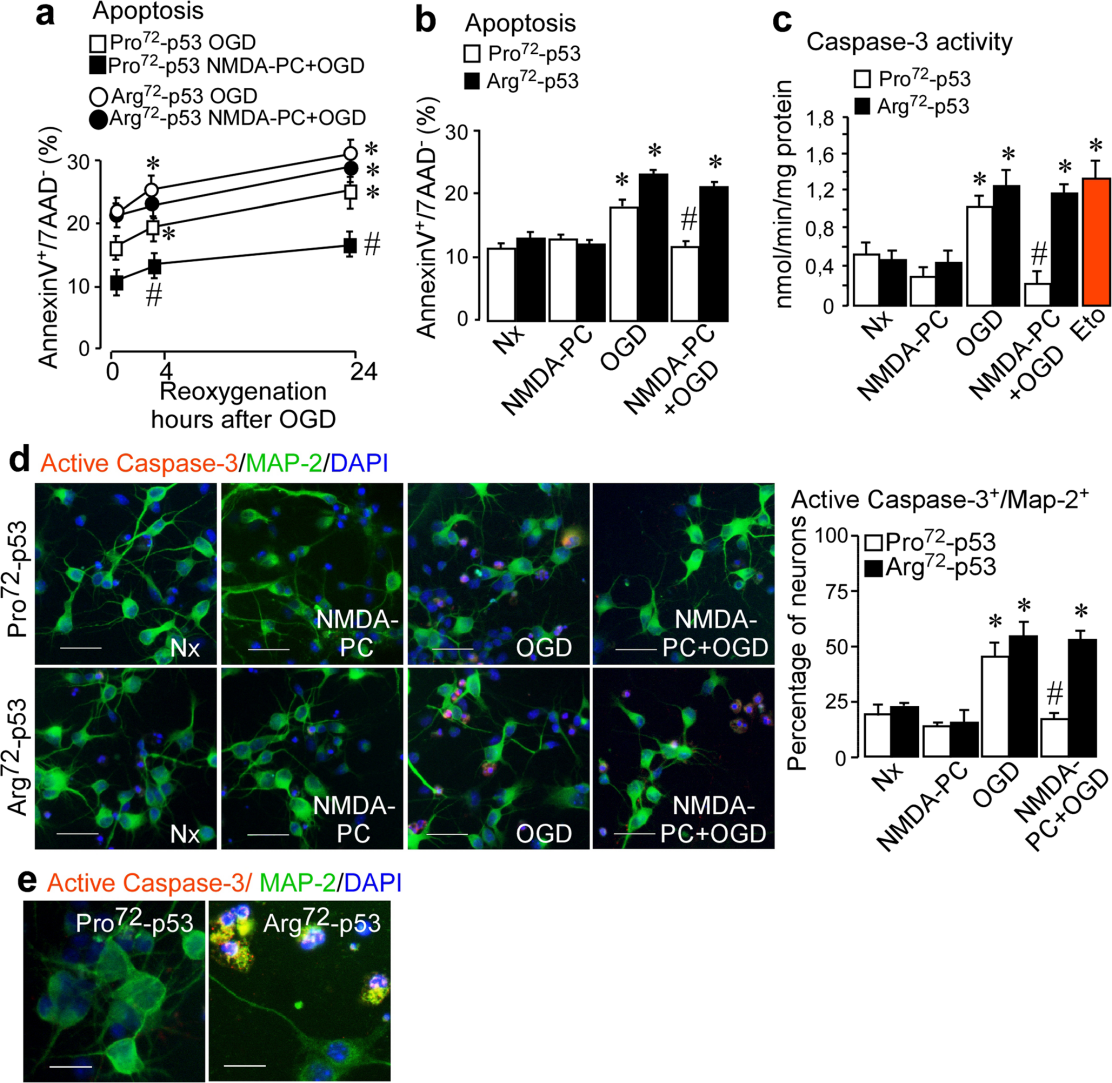
NMDA-PC prevents ischemia-induced apoptosis and promoted caspase-3 activation in Pro72-p53 neurons. Cortical neurons from mice expressing human Pro72-p53 or Arg72-p53 polymorphic variants (9–10 DIV) were exposed to a validated in vitro model of PC. (**a**) At 4 h after OGD time-dependently induced neuronal apoptosis in neurons expressing both human *Arg72Pro* polymorphism variants of p53 (Pro72-p53 or Arg72-p53 neurons), as revealed by flow cytometry. (**b**) This effect was prevented by NMDA-PC in Pro72-p53 neurons, but not in Arg72-p53 neurons. The percentage of annexin V-APC stained neurons that were 7AAD negative were considered to be apoptotic (AnnexinV^+^/7AAD^−^). Accordingly, NMDA-PC also prevented the activation of caspase-3 induced by OGD, as revealed by both fluorimetry assay (**c**) and immunostaining (**d** and **e**) at 4 h after OGD. These results validates the PC method utilized and confirm that preconditioned Pro72-p53 neurons displayed neuroprotection against ischemia. (**d**) Fluorescence microphotographs of both Pro72-p53 and Arg72-p53 neurons were exposed under four conditions Nx, NMDA-PC, OGD, NMDA-PC+OGD (**d**) or under NMDA-PC+OGD (**e**), described previously and after immunostaining for activate caspase-3 (red), MAP-2 (green). Scale bar: 50 μm (**d**) and 25 μm (**e**). Data are means ± S.E.M. (*n* = 3 independent neuronal cultures). Statistical analysis of the results was evaluated by one-way analysis of variance, followed by the least significant difference multiple range test. Student’s *t* test was used for comparisons between two groups of values. In all cases, *p* < 0.05 was considered significant. In all cases *p < 0.05 versus Nx (data not shown in **a**), and #*p* < 0.05 versus OGD

The transcriptional enhancement of pro-apoptotic genes mediated by p53, such as PUMA [44, 45], contributes to activation of caspases and subsequent neuronal death [46]. Having demonstrated that the *Tp53 Arg72Pro* polymorphism controls p53 stabilization after PC, we next studied its relevance in NMDA-PC-induced neuroprotection. Then, we measured apoptotic cell death (Fig. 3a, b) and caspase-3 activation (Fig. 3c) in neurons expressing either Pro72-p53 or Arg72-p53 polymorphic variants. According to our previous results [22], flow cytometry analysis revealed that reoxygenation following OGD promoted apoptosis in a time-dependent manner (Fig. 3a). We also observed that NMDA-PC prevented the ischemia-induced apoptosis in Pro72-p53, after 4 h of reoxygenation (Fig. 3b), which confirmed the neuroprotective effect of controlled NMDA-PC in cortical neurons subjected to ischemia. However, NMDA-PC-induced neuroprotection was not observed in Arg72-p53 neurons. Additionally, OGD induced caspase-3 activation in both genotypes as revealed by fluorimetry assay, which was exclusively prevented by NMDA-PC in Pro72-p53 neurons (Fig. 3c). Furthermore, co-immunostaining with anti-MAP2 (neuronal marker) and anti-active caspase-3 antibodies confirmed the activation of caspase-3 in neurons after OGD, as well as the lower activation of caspase-3 in preconditioned Pro72-p53 neurons, when compared to Arg72-p53 ones after the ischemic insult (Fig. 3d, e). These results validated the PC method utilized and demonstrated the effectiveness of NMDA-PC-promoted control of p53/Caspase-3 pathway in Pro72-p53 neurons, but not in Arg72-p53 neurons.

### TIA Prevents Poor Prognosis After Stroke in Patients with the *Pro72 allele*, but Not in Those with the *Arg*/*Arg* Genotype

**Fig. 4:**
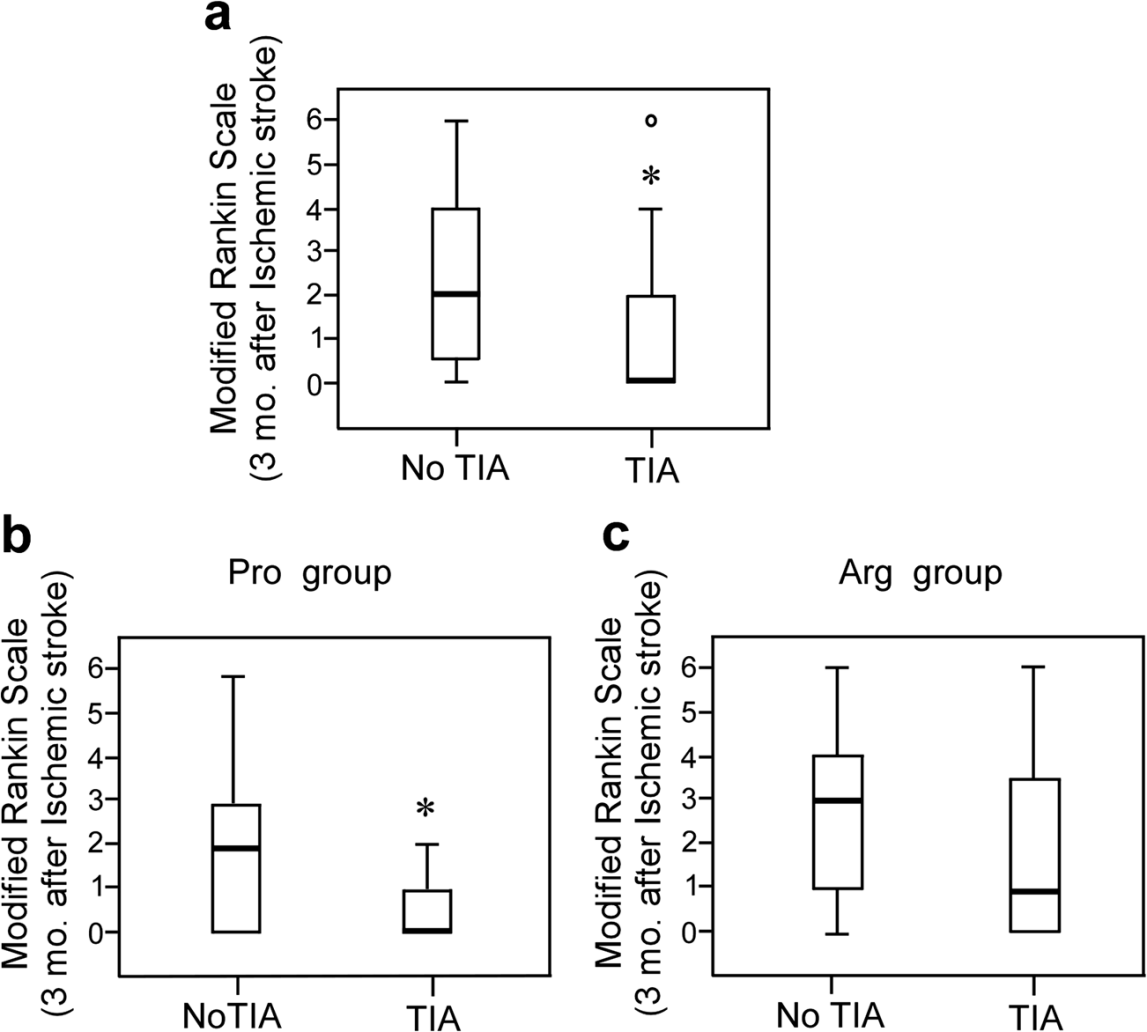
TIA associates with a better prognosis in stroke patients with Pro72-p53 genotype. Eighty-five patients were admitted at the University Hospital of Valladolid, University Hospital of Salamanca and University Hospital Arnau Vilanova of Lleida (Spain). The study included (**a**) 25 TIA patients, (**b**) *Arg*/*Pro* and *Pro*/*Pro*: 13, referred as Pro and (**c**) *Arg*/*Arg*: 12, referred as Arg patients, with TIA within 1 month before stroke and 27 (Pro) and 13 Arg) without TIA, respectively. Modified Rankin Scale (mRS) was used to evaluate the functional outcome of TIA or No TIA patients prior ischemia. mRS score at 3 months after ischemia with indicated *Tp53 Arg72Pro* genotypes (**b**, **c**) or not (**a**). Fisher’s test *p < 0.0001 versus No TIA patients

Finally, to study the possible clinical relevance of our results, we assessed whether the *Tp53 Arg72Pro* SNP was associated with TIA-induced good prognosis after ischemic stroke. We used a cohort of patients with or without TIA within 1 month prior stroke and were matched by functional outcome based on the mRS score [43] (see Table 1 for baseline characteristics). First, we confirm in our cohort that patients suffering a TIA before stroke presented better prognosis at 3 months than patients without previous TIA (Fig. 4a). Next, we study the effects of a TIA within a month prior stroke according to *Tp53 Arg72Pro* patients’ genotype. We found that significant TIA-induced reduction in median mRS scores at 3 months after stroke was observed in patients harboring the dominant *Pro* allele (referred as Pro patients, Fig. 4b), when compared with those not suffering a previous TIA (No TIA) (Fig. 4b). However, this effect was not so evident in patients homozygous *Arg*/*Arg* (referred as Arg patients, Fig. 4c). Moreover, even though TIA also influence the functional outcome of preconditioned Arg patients, 33% of Arg patients exhibited poor prognosis (mRS > 2) after stroke; whereas, the entire group of Pro patients with previous TIA had a favorable outcome (mRS ≤ 2) at 3 months after ischemia stroke (Table 2), suggesting that the *Arg72Pro* SNP modulates the effectiveness of the protective effect exerted by TIA against a subsequent ischemic insult.

**Table 1:** Baseline demographic and clinical features of patients. TIA, transient ischemic attack; NIHSS, National Institute of Health Stroke Scale; SNP, single-nucleotide polymorphism. Patients were admitted at the University Hospital of Salamanca and Valladolid and the University Hospital Arnau de Vilanova, Lleida (Spain). Data are shown as percentage (*n*, %), mean (S. D.) or medians (quartiles)

	TIA (*N* = 25)	No TIA (*N* = 60)
Age (years)	71.20(±13.1)	72.64(±13.1)
Gender		
Males, *n* (%)	14 (56)	33 (55)
Females, *n* (%)	11 (44)	27 (45)
Previous		
Hypertension, *n* (%)	16 (64)	40 (66.6)
Diabetes, *n* (%)	8 (32)	17 (28.3)
Smoking, *n* (%)	7 (28)	8 (13.3)
Hyperlipidemia, *n* (%)	12 (48)	23 (38.3)
Atrial fibrillation, *n* (%)	4 (16)	14 (23.3)
NIHSS on admission		
Minor stroke, *n* (%)	12 (48)	21 (35)
Moderate stroke, *n* (%)	11 (44)	26 (43)
Moderate to severe stroke, *n* (%)	0	12 (20)
Severe stroke, *n* (%)	2 (8)	1 (1.6)
TOAST		
Cardioembolic, *n* (%)	7 (28)	17 (28)
Atherothrombotic, *n* (%)	5 (20)	13 (21.6)
Lacunar, *n* (%)	2 (8)	7 (11,6)
Undetermined, *n* (%)	4 (16)	26 (43.3)
Others, *n* (%)	2 (8)	2 (3.3)
*Tp53 Arg72Pro*		
Arg (*Arg*/*Arg*), *n* (%)	12 (48)	33 (55)
Pro (*Arg*/*Pro* and *Pro*/*Pro*), *n* (%)	13 (52)	27 (45)

**Table 2:** Functional outcome of stroke patients with previous TIA. Patients were matched by good (mRS ≤ 2) and poor (mRS > 2) prognosis at 3 months after stroke with indicated *Tp53 Arg72Pro* genotypes

	Good prognosis	Poor prognosis	*p*
Pro, *n* (%)	13 (100%)	0	0.04
Arg, *n* (%)	8 (67%)	4 (33%)	

## Discussion

Several studies have revealed that polymorphic variants in genes encoding apoptotic proteins could be highly valuable in the diagnosis of stroke [47] and that p53 stabilization contributes to ischemia-induced neuronal apoptosis [19, 48]. Moreover, we recently found that *Tp53 Arg72Pro* SNP controls susceptibility to apoptosis after ischemia and dictates the prognosis of stroke patients [22, 31]. Here, we described for the first time that the human *Tp53 Arg72Pro* SNP has a key function in NMDA-PC-induced neuroprotection and modulates neuronal ischemic tolerance against ischemia-induced apoptosis.

The similar stabilization of p53 reported in Pro72-p53 and Arg72-p53 neurons after OGD, along with unaltered levels of p53 mRNA in ischemic neurons confirms the implication of post-translational mechanism in the ischemia-induced stabilization of p53. In this regard, MDM2 is an essential negative regulator of p53 [49]. MDM2 is an E3 ubiquitin ligase that promotes the rapid and continuous degradation of p53 and modulates p53 localization, stability, and transcriptional activity [49]. We recently found that MDM2-p53 pathway is involved in PC-promoted neuronal ischemic tolerance in both in vitro and in vivo models [30]. More specifically, we have demonstrated that NMDA-PC increases neuronal MDM2 protein levels, thus preventing ischemia-induced p53 stabilization and promoting neuroprotection [30]. Here, we found that neurons subjected to ischemia prior NMDA-PC express high levels of MDM2 in both *Arg72Pro* genotypes. However, the combination of NMDA-PC+OGD prevented the aforementioned stabilization of p53 only in Pro72-p53 neurons indicating that only preconditioned Pro72-p53 neurons might display p53 destabilization and increased protection against ischemia through a MDM2-dependent mechanism. In fact, nuclear accumulation of Pro72-p53 variant might be decisive to MDM2-mediated translocation of p53 from nucleus to cytosol and subsequent degradation by proteasome, which might be responsible for differences between genotypes.

In response to ischemia, a fraction of stabilized p53 translocates to mitochondria leading a rapid pro-apoptotic response in a transcriptional-independent manner [50, 51]. In particular, the mitochondrial accumulation observed in Arg72-p53, but not Pro72-p53 variant, promotes cytochrome c release and caspase 9 activation after ischemia in neurons [22]. Here, we observed that p53 stabilization in mitochondria is not prevented by NMDA-PC in Arg72-p53 neurons, which makes them more vulnerable to ischemia-induced mitochondrial membrane disruption and subsequent apoptotic death with respect to Pro72-p53 ones. This might explain the beneficial neuroprotective effects of NMDA-PC exclusively observed in Pro72-p53 neurons.

The p53 protein controls the cell survival/death decision by inducing apoptosis-related genes, such as PUMA, or promoting caspase activation [21]. Several studies have demonstrated that the absence of p53 and caspase-3 protects neurons against ischemic insult [52]. Here, we show that NMDA-PC prevents ischemia-induced apoptosis and caspase-3 activation in Pro72-p53 neurons, but fails to protect Arg72-p53 cortical neurons against ischemia. Our data are therefore consistent with the idea that the human *Tp53 Arg72Pro* SNP modulates NMDA-PC-promoted neuroprotection against ischemia by controlling the p53 mitochondrial translocation and later p53/caspase-3 pathway.

Transient ischemic attack (TIA) before ischemic stroke could represent a clinical equivalent of cerebral PC [4, 5]. TIAs are temporary episodes associated with relative benign short-term consequences, but they can be a warning signal of an impending stroke. Hence, the relevance of finding an appropriate marker to predict the functional prognosis of patients suffering from TIA might help to act accordingly in case of later stroke. In good agreement with several clinical results [9, 10], we confirmed the neuroprotective role of TIA within first month before an ischemic stroke. Although shorter times between TIA and subsequent stroke could provide better protection, our results showed that 1-month-time interval is also capable to promote this beneficial effect in patients. However, we demonstrated that TIA is not sufficient to ensure better prognosis of patients, but human *Tp53 Arg72Pro* SNP can modulate their evolution. Even though TIA influenced the functional outcome of preconditioned patients, *Arg*/*Arg* genotype associated with poor prognosis after ischemia compared to Pro allele-carrying subjects. The Pro72-p53 variant has been already described as a genetic marker predicting functional outcome after ischemic or hemorrhagic stroke [22]. Here, the benefit associated with Pro patients suffering TIA within first month prior stroke was shown. Taken together, our results indicate that TIA displays a more efficient protective mechanism against poor functional outcome in Pro patients suffering stroke than in Arg patients.

In conclusion, our findings demonstrate that *Tp53 Arg72Pro* polymorphism modulates NMDA-PC-induced neuroprotection against a subsequent ischemic insult, through a mechanism that involves p53 stabilization and modulation of ischemia-induced apoptosis. The clinical relevance of the human *Tp53 Arg72Pro* polymorphism in TIA-promoted neuroprotection and its role in ischemic tolerance are also stablished in patients. Accordingly, the human *Tp53 Arg72Pro* polymorphism might be considered as a noninvasive molecular biomarker to predict the functional prognosis and ischemic tolerance in patients who have experienced a TIA.
