# Correction to: The Neuronal Ischemic Tolerance Is Conditioned by the *Tp53 Arg72Pro* Polymorphism

**DOI:** 10.1007/s12975-026-01417-w

**Published:** 2026-02-03

**Authors:** Maria E. Ramos-Araque, Cristina Rodriguez, Rebeca Vecino, Elisa Cortijo Garcia, Mercedes de Lera Alfonso, Mercedes Sanchez Barba, Laura Colàs-Campàs, Francisco Purroy, Juan F. Arenillas, Angeles Almeida, Maria Delgado-Esteban

**Affiliations:** 1 Institute of Biomedical Research of Salamanca, University Hospital of Salamanca, University of Salamanca, CSIC, Calle Zacarías González 2, Salamanca, 37007 Spain; 2 Institute of Functional Biology and Genomics, University of Salamanca, CSIC, Salamanca, Spain; 3 Stroke Unit, Department of Neurology, University Hospital of Valladolid, University of Valladolid, Valladolid, Spain; 4 Department of Statistics, University Hospital of Salamanca, University of Salamanca, Salamanca, Spain; 5 Clinical Neurosciences Group, IRBLleida. UdL, Lleida, Spain; 6 Stroke Unit, University Hospital Arnau de Vilanova, Lleida, Spain; 7 Neurovascular Research Laboratory (i3), Instituto de Biología y Genética Molecular, Universidad de Valladolid, CSIC, Valladolid, Spain

**Correction to: Translational Stroke Research (2019) 10:204–215**

10.1007/s12975-018-0631-1

During figure assembly, the representative micrograph for Arg72-p53 neurons under normoxia (Nx) in Fig. 3d (active caspase-3/MAP-2 immunostaining) was inadvertently replaced with the image from the Arg72-p53 NMDA preconditioning (NMDA-PC) condition. The figure panel has now been corrected to display the appropriate Arg72-p53 Nx image. This error is limited to the representative image; all underlying data, quantitative analyses, results, and conclusions remain correct and unchanged.

**Original Fig. 3d**:


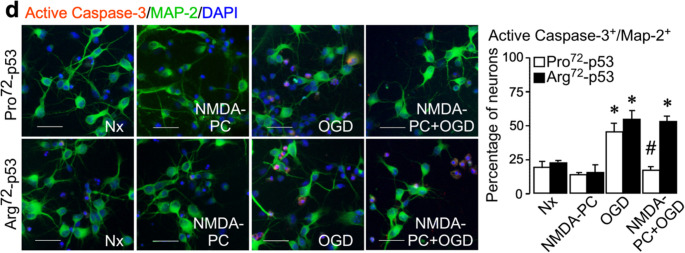


**Corrected Fig. 3d**:


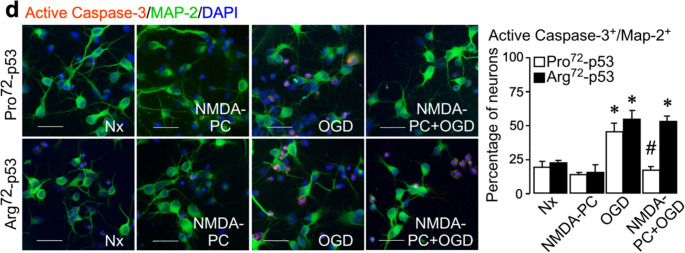


The authors apologize for this error and any confusion it may have caused.

